# Comorbid Insomnia and Obstructive Sleep Apnea (COMISA): Current Concepts of Patient Management

**DOI:** 10.3390/ijerph18179248

**Published:** 2021-09-01

**Authors:** Beatrice Ragnoli, Patrizia Pochetti, Alberto Raie, Mario Malerba

**Affiliations:** 1 Respiratory Unit, S. Andrea Hospital, 13100 Vercelli, Italy; beatrice.ragnoli@aslvc.piemonte.it (B.R.); patrizia.pochetti@aslvc.piemonte.it (P.P.); alberto.raie@aslvc.piemonte.it (A.R.); 2 Department of Traslational Medicine, University of Eastern Piedmont, 28100 Novara, Italy

**Keywords:** OSA, sleep-disordered breathing, COMISA, clinical management

## Abstract

Obstructive sleep apnea (OSA) and insomnia are the two most common sleep disorders among the general population, and they may often coexist in patients with sleep-disordered breathing (SDB). The higher prevalence of insomnia symptoms in patients with OSA (40–60%) compared to that observed in the general population has thus led researchers to identify a new disorder named comorbid insomnia and OSA (COMISA), whose true burden has been so far largely underestimated. The combined treatment of COMISA patients with positive-airway pressure ventilation (PAP) with cognitive behavioral therapy for insomnia (CBT*i*) has shown a better patient outcome compared to that obtained with a single treatment. Furthermore, recent evidence has shown that an innovative patient-centered approach taking into consideration patient characteristics, treatment preferences and accessibility to treatment is recommended to optimize clinical management of COMISA patients. However, in this complex mosaic, many other sleep disorders may overlap with COMISA, so there is an urgent need for further research to fully understand the impact of these therapies on outcomes for OSA patients with comorbidity. In light of this need, this review focuses on the major sleep disorders comorbid with OSA and the recent advances in the management of these insomniac patients.

## 1. Introduction

**Figure 1:**
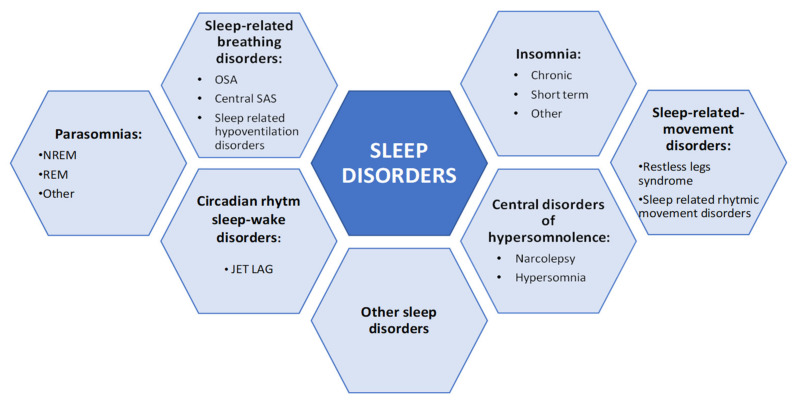
Schematic representation of sleep disorders (ICSD-3). NREM: non-rapid eye movement, REM: rapid eye movement, OSA: obstructive sleep apnea, SAS: sleep apnea syndrome.

Obstructive sleep apnea (OSA) and insomnia are sleep disorders highly represented in general population and that often coexist interacting to amplify an overall greater illness severity [1,2]. The co-occurrence of the two disorders, if not recognized, may complicate OSA treatment reducing compliance with positive airway pressure (PAP) therapy in the presence of insomnia symptoms when not adequately treated together [3,4], and, on the other hand, make insomnia more refractory to cognitive behavioral therapy (CBT*i*) when there is an association with sleep-disordered breathing [5,6]. Recently, researchers have coined the term “COMISA” (Co-Morbid Insomnia and Sleep Apnea) to identify a comorbid condition that can have a worse outcome than the separate conditions and higher management costs [7,8]. The two conditions share some common features and may overlap but have different pathophysiological mechanisms and need a different approach [9]. Unfortunately, this entity has been underestimated for a long time and only recently the interest of the scientific world has focused on recognizing comorbid insomnia and OSA to find an adequate and optimal treatment strategy. Moreover, in this complex framework, many other sleep disorders have been identified that sometimes may overlap with COMISA, making their correct identification, assessment and treatment more difficult. Figure 1 shows the list of diseases that may be diagnosed during sleep according to the International Classification of Sleep Disorders (ICSD) 3rd edition [10].

Given that some of these sleep disorders may occur in the same patients as comorbidities, clinicians should suspect the presence of comorbidities in obstructive sleep apnea (OSA) patients whenever symptoms persist despite adequate treatment. The differential diagnosis is further complicated by the fact that OSA symptoms may often have a different onset from that of the co-occurring sleep disorder, which may also vary over time independently from each other. It is now clear how the main complaint (e.g., OSA or other sleep disorders) needs to be addressed together with the so-called “comorbid” (or secondary) complaint, leading to more specific considerations and an appropriate assessment for a correct management. It is thus important to correctly identify all sleep disorders present in a patient, diagnose them separately and propose an integrated treatment of them to prevent sleep deprivation and preserve sleep integrity and daytime quality of life [11].

## 2. Clinical Features and Relationships

### 2.1. Insomnia and Sleep Apnea

Insomnia and sleep-disordered breathing (SDB) are among the two most frequently diagnosed sleep disorders considering the general population. Because of the high prevalence of co-occurrence of these two conditions, often leading to cumulative morbidity and increased illness severity, a correct and early diagnosis of both disorders is crucial to define their potential interplay and optimize their treatment [12]. In this regard, the first evidence of co-occurrence was reported by Guilleminault et al. in 1973, who described “a new clinical syndrome, sleep apnea with insomnia”, characterized by repeated episodes of apnea occurring during sleep. The augmented breathing in response to apnea was associated with general arousal and often complete awakening, resulting in loss of sleep [13]. The ICSD-3 classification describes insomnia symptoms that may occur comorbidly with another sleep disorder, such as sleep apnea or restless leg syndrome [10] and provides adequate criteria to diagnose comorbid insomnia (Table 1).

**Figure 2:**
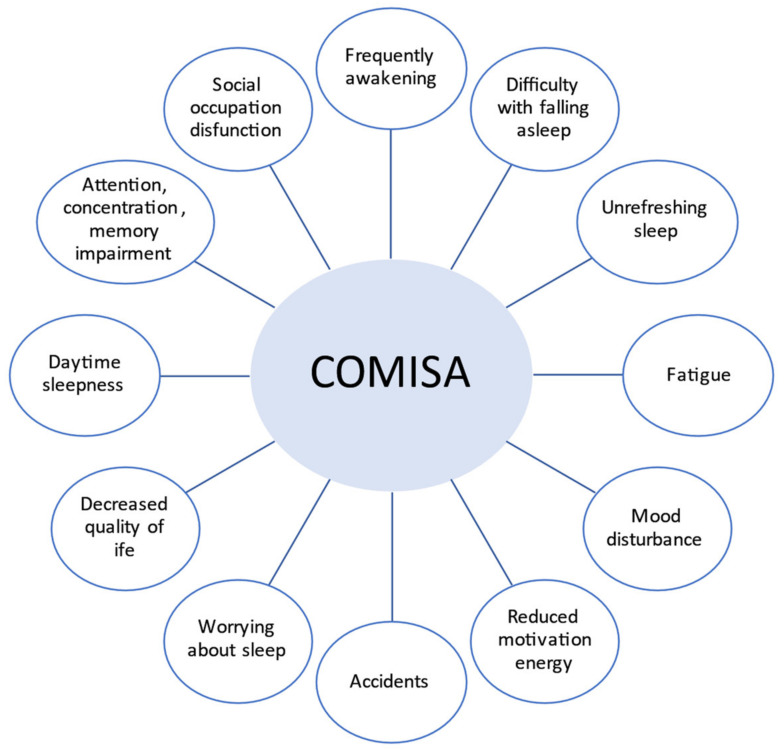
Symptoms of obstructive sleep apnea (OSA) and insomnia (COMISA).

Luyster et al. identified and presented the shared symptoms in both diseases (Figure 2) [14].

According to Ong et al., in individuals with sleep apnea as the primary complaint (e.g., snoring, excessive daytime sleepiness and nocturnal breathing issues), the prevalence of co-occurrence of insomnia was reported as between 6% and 84%. On the other hand, in those patients complaining of the first symptoms of insomnia, the rate of sleep apnea co-occurrence was from 7% to 69% [15]. In addition to clinical features, comorbidity of insomnia and sleep apnea is associated with increased morbidity and impairment. In particular, concurrent insomnia and OSA increase the risk for other conditions [12,15] (Table S1).

One study by Mundt et al., to examine the contributions of OSA and insomnia to chronic pain, found that 80% of participants reported chronic pain, with the most common types of pain being musculoskeletal (28.57%) and headaches (24.76%). A post-hoc comparison showed that individuals with comorbid OSA/insomnia reported an average pain intensity that was 20 points (out of 100) higher than individuals with insomnia or no diagnosis and 28 points higher than those with OSA. They concluded that chronic pain was highly prevalent in this sleep clinic population, with the most severe pain reported by individuals with co-occurring OSA/insomnia with important clinical implications, as both chronic pain and insomnia have been shown to predict poorer adherence to positive airway pressure treatment of OSA [16]. Another retrospective, cross-sectional study by Krell et al. to determine the prevalence of insomnia complaints in patients undergoing evaluation for suspected OSA showed that of 255 consecutive patients, 54.9% reported a complaint of insomnia. Clinical factors associated with insomnia included female gender, psychiatric diagnoses, chronic pain, the absence of regular alcohol use, restless leg symptoms and reports of nocturnal kicking [17].

Furthermore, numerous studies have analyzed a higher risk to develop cardiovascular comorbidities (hypertension, cardiovascular diseases, cerebrovascular diseases, diabetes) among patients presenting comorbid OSA plus insomnia or other sleep disorders [18,19,20,21,22]. An interesting study by Lang et al. examined the prevalence and profile of previously undiagnosed COMISA symptoms (COMISA) in community-dwelling men showing that men with COMISA have a greater prevalence and severity of depression than men with only one disorder [23]. Another piece of research hypothesized that, in OSA patients, sleep onset problems mainly due to hyperarousal and sleep maintenance difficulty primarily relate to sleep-disordered breathing. The authors found different insomnia subtypes that related differently to measures of daytime sleepiness suggesting that OSA patients with sleep onset insomnia may be in a state of hyperarousal. This may be conducive to more appropriate treatment decisions in sleep-disordered breathing [24]. Moreover, some researchers highlighted other conditions related to patients with COMISA, i.e., an increased risk of absenteeism from work [25] and a reduced quality of life with functional impairment [26].

For the aforementioned reasons, it is important to identify all comorbid disorders, diagnose them as separated ones and adequately treat each one of them to avoid long-term sequelae [4].

### 2.2. Patients Phenotypes

**Figure 3:**
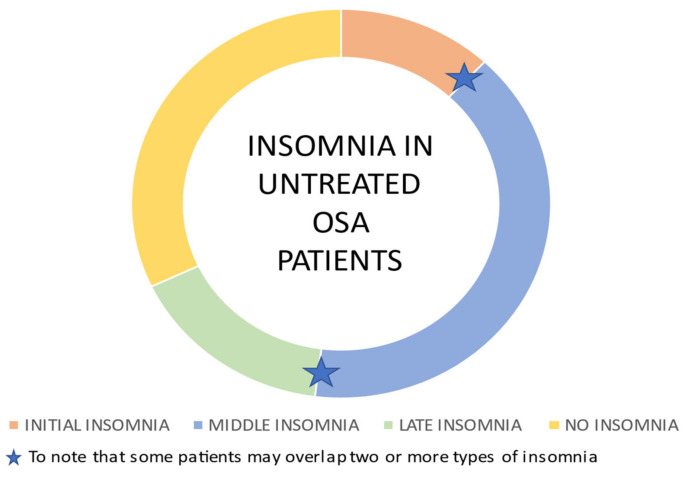
Insomnia subtypes in untreated OSA patients. The majority of untreated OSA patients is affected by middle insomnia consistent with OSA fragmentation induced sleep, while one-third of these patients do not experience insomnia. Some patients refer to difficulty in falling asleep (initial insomnia) or early morning awakenings (late insomnia). Some patients may overlap two or more types of insomnia [4].

Recent research has focused on symptom scores evoking the existence of distinct clinical phenotypes among OSA patients. Among these phenotypes, most patients present sleep disturbances with typical characteristics of chronic insomniac disorder [11]. Indeed, subjects with a primary complaint of OSA are more likely to be female [19,27,28,29,30], to have dysfunctional beliefs about sleep [22,31,32], to suffer from restless legs syndrome [17,33], and to report less alcohol intake compared to OSA-only patients [17]. Conversely, patients with a primary complaint of insomnia are more likely to be male [34,35], to have a higher body mass index (BMI) [36,37], to be older [38,39] and to display more daytime and nighttime nocturnal symptoms consistent with OSA compared to patients without OSA [37,40]. These findings suggest that if a patient’s chief complaint is different to OSA or insomnia, then different clinical considerations, outcomes and treatment responses would need to be considered. For this reason, secondary complaints need to be completely and fully explored [15]. In this regard, an interesting longitudinal cohort study by Bjornsdottir et al. investigated the presence of insomnia symptoms present in OSA patients before being treated for sleep apnea [4]. The authors underline that there are different subtypes of insomnia characterized by difficulties initiating sleep (initial insomnia), difficulties maintaining sleep (middle insomnia) and early morning awakenings (late insomnia) (Figure 3), and they hypothesized that OSA may be a precipitating factor for each subtype of insomnia; on the other hand, insomnia subtypes may be associated with a different level of continuous positive airway pressure (CPAP) adherence to the treatments in OSA patients in a bidirectional relationship.

The results of the study showed that treatment with positive airway pressure (PAP) after two years significantly reduced symptoms of isolated middle insomnia, while other different mechanisms seem to contribute to symptoms of initial and late insomnia in these patients, likely leading to a negative adherence to PAP. This observation suggests the need for targeted treatment for insomnia (i.e., cognitive behavioral therapy for insomnia before PAP treatment) that may be beneficial for patients with OSA comorbid with insomnia affecting positively adherence to PAP. Another subsequent study by Castillo et al. supporting the overlap features between the two comorbid disorders showed how sleep apnea patients who underwent diagnostic polysomnography displayed a large spectrum of total sleep time misperception values, with one-third of the cohort underestimating their total sleep time by at least 60 min [41]. Furthermore, in a recent meta-analysis of Zhang et al., the overall prevalence rates of insomnia, any insomnia complaints, difficulty in falling asleep (DFA), difficulty in maintaining sleep (DMS) and early morning awakening (EMA) recorded in OSA patients were 38%, 36%, 18%, 42%, and 21%, respectively. The authors found that, according to the regional classification of the WHO, the rates of DFA, DMS and EMA among OSA patients in the Western Pacific Region were lower than those in the European Region and the Region of the Americas.

They postulated that the regional differences might have been related to sex, age and BMI [42]. As a general consideration, it is likely to be observed that a deeper investigation of pathogenetic mechanisms is needed and may possibly explain why the coexistence of OSA and insomnia has a negative impact on the two condition’s respective treatment if not promptly recognized.

## 3. Pathogenetic Mechanisms and Models

### 3.1. Systemic Mechanisms

To administer proper treatment, it is also crucial to clarify the pathogenetic mechanisms of the disorders in question, as several mechanisms are believed to play a role in the relationship between OSA and insomnia [43]. Different pathogenetic mechanisms have been proposed to explain the development of comorbid OSA and insomnia. One pathway suggests that OSA may be a risk factor to develop an insomnia disorder. For instance, in sleep apnea associated with cortical arousal, sleep interruptions may be perceived as awakenings or continued wakefulness if these symptoms occur repeatedly [11]. Furthermore, sleep fragmentation caused by apneas may increase anxiety and alertness [44]. PAP treatment in itself has been shown to lead to sleep fragmentation in OSA patients resulting in the development of insomnia. New onset insomnia (NOI), associated with nocturnal ventilatory support, is one of such examples. According to Caetano Mota et al., in patients with OSA under positive airway pressure (PAP) treatment, the prevalence of NOI was 21.4%, and initial and/or intermediate insomnia were the most frequently observed subtypes [45]. Another frequent condition of OSA is nocturia, where patients have to get out of bed several times to go to the bathroom, suffering major sleep disruptions. Some patients may lengthen their time in bed, take periods of rest during daytime and increase caffeine intake to alleviate daytime sleepiness. These behavioral patterns are typical of psychophysiological insomnia, and the countermeasures to alleviate daytime somnolence may then aggravate initiation and maintenance of sleep. Thus, altogether, these findings suggest that nocturnal disturbed breathing is for insomnia [11]. Another pathway proposes a bidirectional relationship between OSA and insomnia. In this regard, the effect of sleep onset on upper airway muscle activity in patients with sleep apnea has been thoroughly investigated. These studies have shown that OSA is a multifactorial disease where non-anatomical features, such as low pharyngeal muscle responsiveness, high loop gain and low arousal threshold may contribute to the pathogenesis of the disease. Muscle activity, in fact, is physiologically reduced in everyone at sleep onset, and its inability to perform the necessary level of neural drive to the upper airway muscles during sleep in response to negative intraluminal airway pressure contributes to the pathogenesis of OSA. Moreover, unstable ventilatory control is characterized by high loop gain likely contributing to cyclical airway obstruction and promoting airway collapse during periods of low ventilatory drive. Finally, low arousal threshold (waking up too easily is likely to contribute to repetitive respiratory nocturnal events) may also have an important role. Upper airway obstruction may be promoted by repeated episodes of awakenings. This effect is greater in OSA patients compared to that seen in healthy subjects [46]. Other models have focused on the potential role of the hypothalamic–pituitary–adrenal axis (HPA) pathway and metabolic factors. It has been hypothesized that stress may increase HPA activity, leading to sleep fragmentation associated with insomnia. In addition, repeated respiratory events (e.g., sleep apneas) can lead to autonomic activation, triggering HPA activation. The increased HPA activity might, in turn, disrupt the metabolic activity associated with metabolic syndrome and glucose imbalance [15]. According to these authors, taking into account the coexistence of cardiovascular and metabolic risk in COMISA, sleep fragmentation may be linked to arousals (a marker of sleep instability) as well as to the activation of HPA and increases in cortisol and metabolic consequences (weight gain, obesity and type 2 diabetes by a potential alteration in timing and amount of food intake, thus impairing glucose tolerance and insulin sensitivity).

### 3.2. Molecular Mechanism

Recent studies support the idea that metabolic processes correlate with sleep; the study of metabolite biomarkers offers unique opportunities to provide insights into the pathology of diseases by revealing alterations in specific metabolic pathways [47]. According to Humer et al., a common pattern in changes of branched-chain amino acid (BCAA), glucose and lipid metabolism, as well as antioxidative status has been identified in patients with sleep-wake disorders; most research has been conducted on OSA where biomarkers related to lipid metabolism seem to be of special importance [47]. In OSA patients, the direct consequence of intermittent hypoxia is an oxidative imbalance with a consequent inflammatory cascade’s activation. Tumor necrosis factor, inflammatory cytokines (IL-2, IL-4, IL-6), lipid peroxidation and cell-free DNA had been found to increase in OSA patients [48]. Reactive oxygen species (ROS) cause damage to the vascular endothelium from the early stage of the disease and stimulate the expression of the adhesion molecules of leukocytes (L-Selectin, integrins) and related endothelial adhesion molecules (Endothelial-Selectin, Platelets-Selectin, Inter Cellular Adhesion Molecule-1, Vascular Cell Adhesion Molecule-1). Endothelial lesions resulting from these biomolecular alterations lead to the microvascular damage of patients with OSA [49]. The repeated cycles of chronic hypoxia/reoxygenation and sleep fragmentation (also in insomnia) that lead to increased ROS production, circulating cytokines and adhesion molecules in OSA patients are correlated to cardiovascular, metabolic and neurodegenerative comorbidities [49].

## 4. Diagnostic Evaluation of COMISA and Clinical Implications

A comprehensive assessment of both OSA and sleep-related disorders is very important to warrant effective treatments, and an integrated, interdisciplinary approach should be considered. For this reason, efficient diagnostic procedures for a combined evaluation are urgently needed. Consequently, the diagnosis of insomnia needs to be enriched by multiple aspects, including a complete patient medical history, several clinical aspects, the results of the physical examination, the recording of sleep diaries and, in selected patients, other complementary techniques, such as actigraphy [50]. On the other hand, polysomnography is for insomnia-related disturbances of sleep initiation and sleep maintenance in the hypnogram [11].

## 5. Current Concepts of Treatment of COMISA

The presence of comorbid OSA, according to Lack et al., does not seem to impair the treatment of insomnia. Following a treatment program for insomnia, the substantial improvement in sleep and daytime functioning variables among COMISA patients was in fact comparable to that observed in patients without OSA [51]. In patients expressing OSA and insomnia complaints, the correct therapeutic strategy seems to be the treatment of both disorders, even though, at the time of writing, no specific and clear guidelines are available to order or sequence of the treatment. Priority should be given—in appropriate multidisciplinary settings—to combined approaches capable of treating patients with comorbid insomnia and OSA [15].

### 5.1. Positive Airway Pressure Devices

The first-line treatment for moderate to severe obstructive sleep apnea syndrome (OSAS) is continuous positive airway pressure (CPAP) [52]. Although treatment of OSA with CPAP provides many health benefits, it is unclear whether this will also improve insomnia for patients with both OSA and insomnia [15]. Bjornsdottir et al. showed that effective CPAP use was associated with a 50% reduction in the prevalence of sleep maintenance insomnia, but symptoms of initial insomnia were largely still present at the two-year follow-up [4]. Patients with OSA and insomnia were less adherent to PAP treatment than those without insomnia, as previously reported [12]. Mendes et al. investigated the effect of nocturnal ventilatory support. In this study, nocturnal ventilatory support was proven effective in treating insomnia secondary to OSAS, with favorable results even in patients that did not meet the criteria for PAP adherence. Furthermore, in the same study, an analysis by insomnia subtype revealed how the majority of patients with mixed insomnia could not overcome the symptoms of insomnia despite an improvement in their apnea-hypopnea index (AHI), sleepiness scale and sleep time [53].

### 5.2. Oral Appliance and Surgery

Other treatments for OSA include the use of mandibular advancement devices (MADs), positional training and surgery. These treatments are typically recommended based on specific features related to sleep apneas (e.g., mild OSA, position-dependent events, and enlarged tonsils) [15]. A recent review of MAD/CPAP treatment in OSA patients has concluded that, despite PAP therapy being superior in reducing AHI, there seem to be no significant differences between the two treatments in terms of daytime sleepiness, cognitive function, vigilance, hypertension and quality of life. Moreover, a randomized crossover trial of veterans diagnosed with OSA reported a significantly greater patient preference and adherence to MAD vs. PAP therapy [54]. According to Guilleminault et al., in selected patients with insomnia and mild OSA, surgery led to a much greater improvement in total sleep time (TST), slow wave sleep and rapid eye movement (REM) sleep duration, respiratory disturbance index (RDI), AHI, minimum oxygen saturation and daytime sleepiness scores. 

### 5.3. Cognitive-Behavioral Therapy for Insomnia

Cognitive and behavioral therapy (CBT*i*) also improved TST and resulted in shorter sleep latency [55]. CBT*i* in the last years has shown a pivotal role in first-line non-pharmacological treatment for insomnia [56,57,58,59,60]. Although CBT*i* is generally regarded as safe and effective across a variety of comorbid conditions, some aspects remain controversial. For example, it has been shown that sleep restriction exacerbates daytime sleepiness due to OSA [11]. According to Ong et al., components of CBT*i* such as stimulus control, aimed at eliminating the extended periods of wakefulness spent in bed, might improve poor sleeping habits in individuals with comorbid insomnia and OSA [15]. In their recent systematic review, Bahr et al. focused on the treatment of patients presenting with both clinical entities of comorbid insomnia and OSA (COMISA) and used a combination of PAP and CBT*i*, or separate treatment alone. Their analysis indicated that almost all the evaluated studies showed a decreased PAP-adherence caused by insomnia. The authors suggested that having comorbid OSA did not impair the treatment of insomnia among COMISA patients. Additionally, insomnia in COMISA patients could be treated with CBT*i* [12]. The overall conclusion of the authors was that both CBT*i* and PAP-therapy are necessary even though distinct treatment strategies may be useful in different COMISA phenotypes.

### 5.4. Benzodiazepine Agents

Even though hypnotics may be used in patients with insomnia, benzodiazepine-based therapies are generally not recommended for patients with OSA [61]. Dolly et al. assessed the effect of fluorazepam on sleep-disordered breathing and nocturnal oxygen desaturation. In their double-blind, placebo-controlled, randomized study, the number of episodes of hypopnea and desaturation did not significantly increase, although the degree of desaturation increased after fluorazepam ingestion. Furthermore, total sleep time significantly increased in fluorazepam-treated patients (*p* = 0.04) [62]. Similar results were obtained by Camacho et al. through a large, randomized trial studying the effect of temazepam on breathing in elderly insomniacs with mild sleep apnea. The study failed to detect any increase in the total number of events (i.e., apnea and hypopnea, and RDI) in elderly subjects with mild OSA receiving 15–30 mg of temazepam [63]. Other authors reported unexpected effects of pharmacological treatment for insomnia. In 1993, Hanly et al. reported that, among central sleep apnea patients, hypnotics not only improved sleep but also decreased apnea frequency, probably by reducing arousals and elevating arterial PCO_2_, improving sleep quality without causing respiratory depression. Consequently, the authors suggested that the statement “hypnotics should not be used in patients with sleep apnea” should be changed to “hypnotics may sometimes be used in patients with sleep apnea” [64]. Two years later, in a randomized double-blind study, Barry et al. assessed the effect of triazolam (0.25 mg) on apnea duration and the arousal response to airway occlusion during sleep in patients with severe OSA. The authors concluded that triazolam increases the arousal threshold to airway occlusion, but this only resulted in a modest prolongation of event duration and increased desaturation [65]. Lastly, given the modest effects of benzodiazepines on OSA, Ong et al. recommended that these drugs should be used with caution in COMISA patients [15].

### 5.5. Non-Benzodiazepine Agents

In recent years, newer non-benzodiazepine sedative hypnotics (NBSH), such as zolpidem, zaleplon and eszopiclone, were developed and studied in patients presenting insomnia with or without concurrent OSA. These compounds refer to non-benzodiazepine (non-BDZ) sedatives acting as BDZ receptor agonists that were shown to have generally a lower harmful impact effect on the airways. According to Luyster et al., non-benzodiazepine agents have hypnotic and sedative effects similar to those of benzodiazepines, but some—especially those selective for gamma aminobutyric acid (GABA) receptors containing α1 subunits—may have fewer muscle-relaxant effects, thereby offering a more favorable treatment approach to insomnia in OSA [14]. In a pilot study by Rosenberg et al. evaluating the acute use of eszopiclone in mild-to-moderate OSA patients, significant differences could not be found in mean scores of respiratory events, total arousals, duration of apnea, hypopnea episodes and oxygen saturation between patients treated with placebo and those treated with eszopiclone. Eszopiclone-treated patient experienced improved sleep duration and sleep efficacy along with reduced wake time during sleep and wake time after sleep onset [65]. Considering the strong and bidirectional association between OSA and insomnia and the observation that zolpidem is one of the most commonly prescribed sedative-hypnotics, recent research tried to determine if this medication may have any effect on the severity of existing sleep-disordered breathing. In a study by Cirignotta et al., a dose of 20 mg of zolpidem, which is above the recommended hypnotic dose, increased the apnea index and oxygen desaturation, suggesting that the usual therapeutic doses of zolpidem should not be exceeded when treating patients with OSA [66]. Berry et al. investigated zolpidem treatment during PAP therapy in OSA patients. The administration of 10 mg of zolpidem to 16 patients with severe OSA that had been treated with an effective level of CPAP did not increase AHI or oxygen desaturation index. Sleep architecture did not differ between zolpidem and placebo nights except for a reduction in sleep latency and mean arousal index during zolpidem nights, indicating that zolpidem does not hinder the efficacy of CPAP in patients with severe OSA [67]. A recent, interesting metanalysis by Nigam et al. assessing the effect of non-benzodiazepines sedative hypnotics on apnea-hypopnea index showed that the majority of patients using NBSH did not develop any worsening of existing apnea-hypopnea index (AHI) regardless of their baseline AHI values (mild, severe, or no OSA) and on the contrary, the use of NBSH in many cases resulted in marginal improvement in baseline AHI when compared to placebo groups [68].

### 5.6. Anti-Depressive Agents

Other authors examined the effects of anti-depressive agents prescribed in OSA patients comorbid with insomnia. Kryger et al. investigated the efficacy and safety of ramelteon in individuals with mild-to-moderate OSA. Ramelteon is a selective melatonin MT(1)/MT(2) receptor agonist indicated for insomnia treatment. The potential effects of ramelteon on apneic and hypopneic events and arterial oxygen saturation in OSA patients was assessed in a randomized, double blind, crossover study, which led to the conclusion that this antidepressant does not worsen sleep apnea when administered to a subject with mild-to-moderate OSA [69]. Decreased serotonergic facilitation of upper airway motor neurons during sleep has been proposed as a risk factor for upper airway obstruction among OSA patients. Serotonin reuptake inhibitors have been shown to produce modest reductions in AHI during non-rapid eye movement (NREM) sleep and sleep fragmentation [70]. Carley et al. showed that 4.5–15 mg of mirtazapine—a mixed 5-hydroxytryptamine receptors (5-HT2/5-HT3) antagonist that promotes serotonin release in the brain—reduced AHI by a half. In their study, mirtazapine was also associated with sedation and body weight side effects in patients with OSA [70]. An emerging theme is that a lower respiratory arousal threshold may be involved in OSA pathogenesis. In this regard, a randomized crossover study by Smales and Malhotra [71] has shown that administration of trazodone, one of the most commonly prescribed agents for treating insomnia, led to a significant reduction of the arousal index and AHI in OSA patients without worsening hypoxemia. Consistently, a systematic review reported no differences in sleep efficiency or rate of discontinuation due to adverse events between trazodone—a dose range of 50 to 150 mg before bedtime—and a placebo in patients diagnosed with chronic insomnia. Although trazodone was more effective in improving subjective sleep quality, there were no differences in sleep onset latency, total sleep time or wake after sleep onset [72].

### 5.7. Sleep Hygiene Education

Sleep hygiene education and pharmacotherapy are the most commonly offered treatments for chronic insomnia disorder. Sleep hygiene education commonly includes information about caffeine, alcohol and nicotine use, exercise, sleep environment, sleep–wake regularity, nap avoidance and stress management [73]. A recent study by Jung et al. including patients with sleep apnea showed that among the factors of sleep hygiene-related conditions, inadequate temperature and humidity, drinking alcohol before sleep and emotional excitement or arousal were associated with symptoms of mild to moderate OSA. This study supports the hypothesis that patients with mild to moderate OSA can experience symptom relief if they are trained to correct lifestyle habits to maintain adequate sleep hygiene-related conditions [74].

### 5.8. Melatonin and Herbal Therapies

A meta-analysis on the use of melatonin for treating primary sleep disorders showed an approximately 7-min decrease in sleep latency, an 8-min increase in total sleep time and a very small improvement in sleep quality. Adverse events were not discussed [75]. Likewise, the use of valerian and chamomile for the treatment of insomnia has not been supported by evidence. Specifically, in a systematic review, no differences in either daytime functioning or insomnia severity were found for valerian or chamomile vs. placebo [76]. Melatonin may be a therapeutic option in COMISA comorbid with circadian rhythm sleep–wake disorders [77].

## 6. COMISA and Other Sleep Disorders

According to Figure 1, many other sleep disorders were identified, and some of them may overlap with COMISA.

### 6.1. Sleep Related Movement Disorders

Restless legs syndrome (RLS) patients may show insomnia-specific characteristics such as sleep disruptive habits and cognitions [78], and similar data were found in patients suffering from sleep apnea [79]. Compared with patients who have just sleep apnea syndrome, those with both OSA and RLS were shown to exhibit a higher degree of insomnia-specific psychological symptoms, which may indicate the benefit of cognitive behavioral therapy in these populations [80]. In previous studies, arousal index, periodic leg movements (PLM) index and PLM-arousal index were more pronounced in patients with OSA and RLS, showing that RLS in OSA patients is an independent risk factor for difficulties in sleep maintenance [80,81]. Moreover, CPAP therapy may influence PLM indices in OSA patients [82]. The question of whether disturbed sleep quality in these patients is caused by RLS, OSA or an insomnia disorder cannot easily be cleared; however, the fact that insomnia comorbid with other sleep disorders should be treated with insomnia-specific methods is an important feature of insomnia management [83].

### 6.2. Epilepsy and Parasomnias

There is increasing evidence that obstructive sleep apnea with insomnia symptoms coexist in epilepsy (in 10% of unselected adult epilepsy patients, up to 30% of drug-resistant epilepsy patients). Continuous positive airway pressure treatment of OSA in epilepsy improves seizure control, cognitive performance and quality of life [84]. Parasomnias and epileptic seizures can coexist in the same subject, making the differential diagnosis particularly challenging: REM Sleep Behavior Disorder (a REM-sleep parasomnia) has been found to co-occur in 12% of elderly epilepsy patients, and a particular pattern of association has been found between nocturnal frontal lobe epilepsy (NFLE) and NREM arousal parasomnias [85]. Patients with epilepsy often complain of poor, non-restorative sleep; however, insomnia in epilepsy is poorly investigated, and sleep hygiene measures in epilepsy need to be more comprehensive, taking into account the various pathologies that may underlie sleep disorders in epilepsy patients [86].

### 6.3. Central Disorders: Narcolepsy

OSA occurs frequently in narcolepsy and may delay the diagnosis and interfere with narcolepsy proper management. In patients with OSA, cataplexy (a major narcoleptic trait) should be actively looked for to exclude the presence of narcolepsy. OSA and narcolepsy are associated with poor quality of sleep and excessive daytime sleepiness (EDS): treatment with CPAP does not usually improve EDS in narcoleptics with OSA [87]. A new medication, Solriamfetol, belonging to the class of dopamine-norepinephrine reuptake inhibitors (DNRI), has recently received FDA approval to treat excessive daytime sleepiness in patients with abnormal sleep patterns, such as obstructive sleep apnea (OSA) and narcolepsy [88]. The approval was supported by data from the phase 3 TONES (Treatment of Obstructive sleep apnea and Narcolepsy Excessive Sleepiness) clinical program, which included five randomized placebo-controlled studies [89,90,91]. All trials showed superiority of Solriamfetol relative to placebo even in subgroups of patients adherent or nonadherent to OSA treatment [92]. Common side effects were also recently investigated and reported by Rosenberg et al. and may include headache, nausea, decreased appetite, insomnia and anxiety [93].

### 6.4. Circadian Rhythm Disorders

Circadian rhythm sleep–wake disorders are a collection of disorders that can occur when patients’ typical sleep times are misaligned. Many people have experienced jet lag, travelling across at least three time zones, or shift work sleep phase disorder, affecting those who have a non-traditional work schedule, resulting in poor sleep and exhaustion during waking hours. It is important to consider whether another sleep disorder might be occurring, such as sleep apnea, that may be exacerbated and associated with new-onset insomnia. Therapeutic options include CBT*i* with progressive delay of the circadian rhythm with the strategic use of light therapy, natural light exposure and melatonin [77]. 

## 7. Implications of the Findings and Future Research

OSA is the most common kind of sleep apnea characterized by upper airway collapse during sleep with repeated episodes of apneas and hypopneas leading to oxygen desaturations; it is related to many other medical conditions such as congestive heart failure, hypertension, stroke, arrhythmia and decline in renal function [94] that may have a negative prognostic effect on long-term survival as demonstrated in our unit in a retrospective observational cohort study recently conducted [95]. At the same time, OSA can be associated with very frequent sleep disorder comorbidities, i.e., insomnia. Both OSA and insomnia are highly prevalent in the general population. They share some clinical features and may aggravate each other because of reciprocally adverse pathogenetic mechanism, which makes COMISA a clinically relevant condition requiring an integrated and multidisciplinary approach [11]. The variability in the definition of insomnia and OSA in available studies make it difficult to determine the effective overlap of these conditions. Insomnia symptoms may be present in OSA patients at baseline or appear during treatment. In insomnia patients with maintenance problems and low efficacy of CBT*i* and hypnotics, it is recommended to evaluate signs and symptoms of OSA [54]. In this optic, more punctual phenotyping of clinical and polysomnographic features is needed. Among pharmacological therapies, the newer non-benzodiazepine agents, such as zolpidem and eszopiclone, seem to have a less deleterious effect on the airway. Randomized studies seem to indicate that zolpidem does not impede CPAP efficacy in patients with severe OSA [67]. Thus, clinical judgment should guide whether to start with PAP therapy—or another suitable option for OSA—or CBT for insomnia, or both [11,15]. In this regard, a recent randomized trial has shown that combined treatment of COMISA patients with CBT*i* and CPAP is more effective in improving CPAP use and insomnia symptoms compared to treatment with CPAP alone [96]. Fittingly, treating both insomnia and OSA provides the best outcome for COMISA patients [55]. For this reason, a multidisciplinary, patient-centered approach is recommended to optimize the clinical management of COMISA [97].

## 8. Conclusions

The evidence summarized in this review indicates that the association between insomnia and OSA is an important, yet unexplored, research area that needs to be studied in depth. Recent research interest has focused on the idea of better management with a multidisciplinary approach and concurrent treatment of the two entities that seems to be promising but needs additional data to determine the effectiveness of combination treatment in this population. Globally, it is advisable to look for comorbidities in OSA patients when their symptoms persist despite adequate treatment. Another important aspect emerging from our study is that the onset of OSA symptoms may be independent from that of other co-occurring sleep disorders. In light of this complex comorbidity profile in OSA patients, it is highly recommended for clinicians to investigate the potential co-occurrence of other sleep disorders, diagnose them as comorbidities and treat them accordingly to preserve sleep integrity and improve daytime quality of life.

## Figures and Tables

**Table 1:** Diagnostic criteria for comorbid Insomnia (ICSD-3).

**A Chronic Insomnia Disorder Diagnosis Would Apply Only When:**
The insomnia symptoms show some independence in their onset or variation over time from the other symptoms of the co-occurring sleep disorder. When insomnia symptoms persist despite marked symptom improvement of the co-occurring sleep disorder following adequate treatment. A chronic insomnia disorder diagnosis would not apply when effective treatment of the coincident sleep disorder resolves the insomnia symptoms.

## Supplementary Materials

The following are available online at www.mdpi.com/article/10.3390/ijerph18179248/s1, Table S1: Main comorbidities of COMISA and other sleep disorders. References [16,17,18,19,20,21,22,23,24] are cited in the supplementary materials.
