# Supplementary material for: Comorbid Insomnia and Obstructive Sleep Apnea (COMISA): Current Concepts of Patient Management
Source: Int J Environ Res Public Health. 2021 Sep 1;18(17):9248. doi: 10.3390/ijerph18179248 (PMC8430469; doi:10.3390/ijerph18179248)
Supplement: Supplementary file 1 [file ijerph-18-09248-s001.zip › ijerph-1329595-supplementary.pdf]

Supplementary Materials

Table S1. Main comorbidities of COMISA and other sleep disorders.

Authors	Sample Size	Comorbidity	Main Findings
Mundt J. (2017)<br>[16]	OSA patients<br>*n* = 105	Chronic pain	• Muscoloskeletal pain (28.37%)<br>• Headaches (24.76%)<br>Post-hoc comparisons in COMISA patients showed an average pain intensity that was:<br>- 20 points (out of 100) higher than individuals with insomnia or no diagnosis<br>- 28 points higher than those with OSA
Krell S.B. (2005)<br>[17]	Clinically suspected OSA patients<br>*n* = 255	Chronic pain	Clinical features associated with insomnia included:<br>- Female gender<br>- Chronic pain
Lin C.-L. (2017)<br>[18]	SD patients<br>*n* = 15.109<br>+<br>nSD control cohort<br>*n* = 29.955	Hypertension	Patients with Sleep Disorders (SD) had a higher risk for developing hypertension compared with nSD cohort, particularly pronounced among those who were younger adults (age 40 years) and who had insomnia.
Vozoris N.T. (2012)<br>[19]	Patients<br>*n* = 12.593<br>OSA (*n* = 236/546)<br>Non OSA<br>(*n* = 3550/12,047)	Hypertension<br>Cardiovascular diseases	Individuals with sleep-apnea-plus (similar to sleep-apnoea alones) have elevated rates of cardiovascular (CV) diseases when compared to general population even without reaching a statistically significance.
Meira e Cruz (2021)<br>[20]	Patients<br>*n* = 685 with suspected COMISA	Hypertension<br>Cardiovascular diseases<br>Diabetes	Mild, moderate and high risk for COMISA present increased frequency of associated comorbidities (CVD, systemic arterial hypertension, diabetes)

Gupta (2014) [21]	OSA Patients<br>$n = 7234$<br>of which 658 OSA+I	Hypertension<br>Cerebrovascular disease	In a nationally representative sample, OSA+I was significantly more frequently associated with essential hypertension.
Cho (2018) [22]	OSA Patients<br>$n = 476$<br>of which 139 OSA+I	Cardiovascular disease	High prevalence of Comorbid insomnia with OSA (29.2%). COMISA is a cumulative risk factor for cardiovascular disease. OSA+I group show lower QoL, lower quality of sleep, higher sleep propensity and higher depression.
Lang C.J. (2017) [23]	Men<br>$n = 700$	Depression	Men with COMISA have a greater prevalence, and severity of depression than men with only one disorder.
Chung K.F. (2005) [24]	OSA patients<br>$n = 157$	Sleepiness	There were significant inverse relationships between sleep onset insomnia and measures of daytime sleepiness.<br>On the contrary, subjects with repeated wakening had more severe subjective sleepiness.

COMISA: Comorbid insomnia and OSA, SD: sleep disorders, nSD: non sleep disorders, CVD: cardiovascular disease, OSA+I: OSA plus Insomnia, QoL: quality of Life.
